# Detecting acute kidney injury in horses by measuring the concentration of symmetric dimethylarginine in serum

**DOI:** 10.1186/s13028-021-00568-0

**Published:** 2021-01-15

**Authors:** Natalia Siwinska, Agnieszka Zak, Urszula Paslawska

**Affiliations:** 1 Department of Internal Medicine and Clinic of Diseases of Horses, Dogs and Cats, Faculty of Veterinary Medicine, Wroclaw University of Environmental and Life Sciences, C.K. Norwida 25, 50-375 Wroclaw, Poland; 2 Department of Immunology, Pathophysiology and Veterinary Preventive Medicine, University of Environmental and Life Sciences, C.K. Norwida 25, 50-375 Wroclaw, Poland; 3 Veterinary Institute, Faculty of Biological and Veterinary Sciences, Nicolaus Copernicus University, Torun ul. Gagarina 7, 87-100 Torun, Poland

**Keywords:** Acute kidney injury, Gentamicin, Nephrotoxicity, NSAIDs, SDMA

## Abstract

**Background:**

Acute kidney injury (AKI) in horses may develop as a complication of a primary disease or following the administration of nephrotoxic drugs, and may pose a diagnostic challenge. Hence, the main objective of this study was to evaluate the concentrations and diagnostic significance of serum symmetric dimethylarginine (SDMA) and conventional renal dysfunction biomarkers in healthy horses, horses at risk of developing AKI, and horses with clinically evident AKI. A second aim was to assess how gastrointestinal disease and exposure to potentially nephrotoxic drugs affected SDMA levels. Thirty healthy horses, 30 horses with gastrointestinal disease and/or receiving phenylbutazone or gentamicin (risk group) and 11 horses with AKI were included in the study. Serum SDMA levels were measured using commercially available enzyme immunoassay tests.

**Results:**

SDMA levels in healthy horses, horses at risk of AKI and horses with AKI were 12 µg/dL (11–14), 12 µg/dL (11–13) and 20 µg/dL (20–37), respectively (all results presented as a median (quartile 1–quartile 3)). There was a significant difference in SDMA concentration between the healthy horses and those with AKI, whereas the SDMA levels in healthy horses and those at risk of AKI were comparable. A SDMA cut-off value of 19 µg/dL was established. Horses from the risk group had higher urine protein concentration and urine protein to creatinine ratio compared with healthy horses. Furthermore, horses with colic from the risk group presented with elevated urine γ-glutamyl transpeptidase to creatinine ratio.

**Conclusion:**

The SDMA cut-off value established in healthy horses was higher than previously reported. The SDMA level correlated with the azotaemia levels. Horses from the AKI risk group had normal SDMA levels but single urine parameters was abnormal indicating their higher sensitivity in assessing subclinical kidney dysfunction.

## Background

Acute kidney injury (AKI) is a clinical syndrome presenting as a rapid decline in kidney function due to both structural damage and functional impairment, which often develops as a complication of a primary disease [1, 2]. Early diagnosis poses a challenge as AKI often has a subclinical manifestation at early stages with only non-specific changes in the urine, such as hyper- or hyposthenuria, changes in urine electrolyte clearance or enzymuria. Ultrasound imaging is either inconclusive or shows subtle changes, such as renal enlargement, perirenal oedema, or hyperechoic renal cortex [3, 4]. Unlike renal failure, which often progresses to severe azotaemia, AKI may represent subclinical injury with or without functional impairment, resulting in no or only a slight change in serum creatinine levels. However, as shown in human studies, even mild AKI with minor symptoms can lead to severe clinical consequences [1, 2, 5]. The prevalence of AKI in horses has been demonstrated to be equal to that in humans and companion animals–especially in cases admitted to intensive care units [6, 7]. In horses, AKI predisposing factors include haemodynamic changes, such as dehydration or hypovolaemia, endotoxaemia (e.g. associated with gastrointestinal disorders) and the use of potentially nephrotoxic drugs, e.g. aminoglycosides and nonsteroidal anti-inflammatory drugs (NSAIDs) [7–14].

Currently, the diagnosis of AKI in horses is mainly based on a finding of elevated serum creatinine concentration. However, serum creatinine levels only increase at a relatively late stage, i.e., when around 50% of kidneys function has already been lost. Furthermore, a number of non-renal factors can additionally affect creatinine levels [15]. Therefore, it is necessary to identify sensitive and specific diagnostic markers of equine AKI.

Studies in horses have attempted to evaluate renal function by analysis of the fractional excretion of sodium (FENa), urine γ-glutamyl transpeptidase to creatinine ratio (GGT:Crea), as well as cystatin C and neutrophil gelatinase-associated lipocalin (NGAL) levels, but with inconsistent results [8, 16–18]. Studies in human and companion animals, on the other hand, assessed multiple serum and urinary parameters as potential AKI biomarkers, symmetric dimethylarginine (SDMA) being the most common [19–25]. SDMA levels have previously been determined in healthy adult horses and in neonatal foals [26–28] but only one study has assessed SDMA in horses with kidney dysfunction; however a different diagnostic test was used [29].

The main objective of our study was to determine serum SDMA levels alongside the more conventional renal dysfunction biomarkers as well as their diagnostic value in horses with clinically manifest AKI compared to healthy horses and horses at high risk of developing AKI. The second objective was to determine potential correlations between SDMA levels and levels of more conventional renal dysfunction biomarkers, as well as to investigate how gastrointestinal disease and exposure to potentially nephrotoxic drugs affected various biomarker levels.

## Methods

Samples collected for as part of another study were used. A detailed description of study groups and additional results have been previously published [30].

### Animals

The study was carried out in 71 adult warm-blood horses of different breeds (15 Polish Half-bloods, 13 Lesser Poland, 8 Hanoverian, 8 Thoroughbred, 8 Silesian, 6 Arabian, 6 Holstein, 4 Greater Poland, 3 KWPN Dutch) and of different sex and age. The animals were divided into three groups: the “non-AKI group” of healthy horses; the “high-risk group” consisting of horses at high risk of developing AKI without any evidence of clinical kidney dysfunction and the “AKI group” of horses with clinically manifest AKI. All animals were patients of the equine clinic at the Wroclaw University of Environmental and Life Sciences. Previous medical history of each horse was obtained from all the owners. The body weight was determined using a measuring tape and a horse scale in stabled horses and horses seen at the clinic, respectively. All horses underwent a full clinical examination, blood analysis, urinalysis including sediment assessment and urinary tract ultrasound (transabdominal in all horses, with an additional transrectal ultrasound in some high-risk horses and all AKI horses).

The non-AKI group consisted of 30 healthy horses (15 mares and 15 geldings) between 3 and 29 years of age (mean age 14.7 y; standard deviations (SD) ± 8.4) with a mean weight of 554 ± 80) kg. The inclusion criteria were: (1) a good body condition score (4 or 5 on a 9-point scale), (2) no past or present history of urinary or cardiovascular disease, (3) no clinical signs of local or systemic disease on enrolment and no history of such illness within at least 6 months prior to enrolment and after study completion, (4) haematology, serum biochemistry and urinalysis results within the reference range, (5) no abnormal renal findings on transabdominal ultrasonography, (6) no medical treatment within 6 months prior to enrolment. Each animal was assessed at 6-month intervals during routine veterinary examinations and observed daily by their owners.

The high-risk group consisted of 30 horses (15 mares and 15 geldings) between 2 and 27 years of age (mean age 14.7 ± 7.6 y) with a mean weight of 494 ± 67 kg. Three subsets (10 horses each) were identified within the high-risk group. Subset I consisted of horses treated for colic/gastrointestinal diseases (5 with large colon impaction, 2 with left dorsal displacement of colon, 2 with sand colic, and 1 with right dorsal colon displacement), four of which underwent laparotomy. Subset II consisted of horses treated with oral phenylbutazone (2.2 mg/kg b.i.d.) for 10 days, whilst subset III consisted of horses treated with intravenous gentamicin (6.6 mg/kg s.i.d.) for 5 days. Phenylbutazone and gentamicin were prescribed for a number of indications, such as orthopaedic problems (n = 8), traumatic wounds (n = 7) and upper and/or lower respiratory infection (n = 5). There was no case of systemic manifestation, such as endotoxaemia, sepsis, or a systemic inflammatory response syndrome (SIRS), secondary to primary infection in horses from the high-risk group throughout the entire hospitalisation period. Serum creatinine levels in this group were required to be within the reference range on admission and could only be slightly elevated after treatment (on study visit), not exceeding 25% above the baseline [31].

The AKI group consisted of 11 horses (6 mares and 5 geldings; 2–20 years old (mean age 9.4 y; SD 5.8) with a mean weight of 480 ± 61) kg) with clinically manifest AKI. They were referred to the University Equine Clinic with their primary disease and were diagnosed with AKI on admission. The inclusion criteria were primary acute disease accompanied by azotaemia (serum creatinine level > 159 µmol/L and blood urea nitrogen (BUN) > 6.7 mmol/L). The presence of kidney dysfunction in this group was confirmed based on (1) abnormal urinalysis findings (isosthenuria, proteinuria, elevated FENa and GGT:Crea), and/or (2) abnormal kidney ultrasound findings (perirenal oedema, n = 3; increased corticomedullary differentiation, n = 2; renal enlargement, n = 2). AKI developed in this group secondary to various primary conditions (cardiovascular impairment, n = 4; treatment with potentially nephrotoxic drugs, n = 4; endotoxaemia, n = 2; post-anaesthetic rhabdomyolysis, n = 1). The exclusion criteria were presence or a history of urinary tract disease (e.g., congenital urinary tract anomalies; nephrolithiasis, ureterolithiasis, urolithiasis; neoplasms; bladder paralysis; chronic kidney disease), isolated elevated serum creatinine level in the absence of other signs of a primary disease.

All examinations performed as a part of this study were non-invasive and formed a part of a routine diagnostic assessment. All blood and urine samples were previously utilised in another study. Therefore, some data (i.e. clinical assessment, blood analysis and urinalysis) are duplicated [30].

### Sampling

Blood was collected from the jugular vein with a 20G needle and a 20 mL syringe and put into 2 mL EDTA tubes (Medan, Poland) and 10 mL tubes with a clot activator (Medan, Poland). Urine samples were collected into sterile containers during spontaneous urination in all horses, except oliguric animals and those undergoing laparotomy, where urine samples were collected during aseptic catheterisation. Blood and urine needed for SDMA and conventional kidney dysfunction biomarker assays were sampled once from each horse. In the high-risk group, samples were collected at different time points throughout the study, depending on the subset. In horses with gastrointestinal disease, samples were collected on admission and before inpatient treatment. In animals treated with gentamicin/NSAIDs, the samples were collected following treatment completion. In horses with AKI, samples were collected on inpatient admission. Referred horses from high-risk and AKI groups had serum creatinine and BUN levels determined by the referring practice prior to enrolment.

### Blood analysis

The haematology assays were performed in an EDTA blood sample using a Scil Vet ABC animal blood counter (Horiba, USA) in order to determine the red blood cell (RBC), white blood cell (WBC) counts as well as haemoglobin and haematocrit levels. The blood and clot activator were centrifuged. The serum was divided and placed in 1 mL Eppendorf tubes (Eppendorf, Germany). Fresh serum samples were immediately transported to an external veterinary laboratory for biochemical analysis carried out using the AU680 analyser (Beckman Coulter, CA, USA) and dedicated reagents in order to determine serum creatinine, BUN, aspartate aminotransferase (AST), alanine aminotransferase (ALT), GGT, alkaline phosphatase, creatine kinase (CK), total protein (TP), albumin, glucose, sodium, potassium, chloride, total magnesium and total calcium levels. One of the Eppendorf tubes with serum was refrigerated and transported to an external veterinary laboratory for an SDMA assay.

### Urinalysis

All urine samples were evaluated for colour, clarity, specific gravity (SG), pH, as well as the presence of protein, creatinine, glucose, blood, acetone, urobilinogen, and bilirubin. Urinary GGT and sodium concentrations were assessed using the AU680 chemistry analyser (Beckman Coulter, CA, USA) and dedicated reagents. The urine protein to creatinine ratio (UPCR) and urine GGT:Crea were also determined. Fractional sodium excretion (FENa) was calculated based on the obtained results as per the following formula: [FENa (%) = 100 × (urinary sodium level × serum creatinine/serum sodium × urinary creatinine)].

### SDMA analysis

All serum SDMA assays were carried out in an external veterinary laboratory (IDEXX GmbH, Ludwigsburg, Germany). The SDMA level was determined using a validated, commercially available enzyme immunoassay (EIA) on the Beckmann Coulter analyser (CA, USA).

### Statistical analysis

The normality of distribution assumption was verified using the Shapiro–Wilk test. Where the normality of distribution assumption was not violated, one-way ANOVA with Fisher’s LSD post-hoc test was used for comparisons. All normally distributed variables were presented as means ± standard deviations (SD), while all non-normally distributed variables were presented as medians (Me) and quartiles (Q1–Q3). All between-group differences (non-AKI vs. high-risk (and subsets) vs. AKI group) in quantitative variables were determined using the Kruskal–Wallis test with the post-hoc Conover pairwise comparison further adjusted by the Benjamini–Hochberg method. The correlations between the selected serum and urine parameters and SDMA levels were determined using the Spearman’s correlation coefficient. A Receiver Operating Characteristic (ROC) curve was plotted to determine the sensitivity, specificity, as well as both the positive (PPV) and negative predictive value (NPV) of SDMA assay in detecting acute kidney injury. The P < 0.05 was considered significant for all comparisons. All analyses were performed using the R software for Windows (version 3.6.1).

## Results

The results of the basic blood assays have been published previously [30]. Table 1 shows the selected serum and urine parameters in different groups of animals alongside the number of animals with abnormal findings. The non-AKI and high-risk horses had significantly lower serum creatinine and BUN levels, as well as higher urinary pH than horses with AKI. Horses with AKI, however, had significantly higher urinary protein levels as well as UPCR and GGT than non-AKI horses. Both non-AKI and high-risk horses treated with NSAIDs had significantly lower FENa levels than horses with AKI. In the high-risk group, animals with colic and those treated with NSAIDs had significantly higher urinary protein levels and UPCR than non-AKI horses. Furthermore, animals with colic had significantly higher GGT:Crea ratio than non-AKI horses.

**Table 1:** Selected serum and urine parameters measured in healthy horses (non-AKI group), horses at risk of acute kidney injury (high-risk group; divided into horses with colic and horses treated with NSAIDs and gentamicin), and horses with clinically manifest acute kidney injury (AKI group)

Parameter		Non-AKI group (n = 30)	High-risk group (n = 30)								AKI group (n = 11)	n’
			Colic (n = 10)	n’	NSAIDs (n = 10)	n’	Gentamicin (n = 10)	n’	All	n’		
		Me (Q1–Q3)	Me (Q1–Q3)		Me (Q1–Q3)		Me (Q1–Q3)		Me (Q1–Q3)		Me (Q1–Q3)	
Serum	Urea (3.3–6.7 mmol/L)	5.7 (4.6–6.3)	5.9 (5.4–6.4)	0	5.4 (5.2–5.7)	0	6.2 (5.6–6.4)	0	5.7 (5.3–6.4)	0	12.3^ac^ (10.6–23.9)	11
	Creatinine (71–159 µmol/L)	120.0 (93.0–137.0)	125.1 (105.6–144.5)	1	133.5 (124.0–155.0)	1	124.9 (99.3–129.7)	0	129 (103.8–136.9)	2	305.0^ac^ (228.0–440.2)	11
Urine	pH (7.5–8.5)	8.0 (8.0–8.4)	7.75 (7.5–8.07)	2	8.5 (7.96–8.5)	2	8.0 (7.9–8.5)	0	8.0 (7.8–8.5)	4	6.0^ac^ (5.45–7.95)	5
	SG (1.02–1.04 g/L)	1.031 (1.021–1.042)	1.031 (1.021–1.039)	0	1.03 (1.019–1.034)	1	1.035 (1.031–1.036)	0	1.033 (1.02–1.036)	1	1.013 (1.011–1.022)	4
	Protein (< 1 g/L)	0.07 (0.03–0.1)	0.3^b^ (0.23–1.16)	3	0.21^b^ (0.14–0.24)	1	0.14^c^ (0.09–0.16)	0	0.21^b^ (0.13–0.32)	4	0.69^a^ (0.38–1.24)	5
	UPCR (< 0.5)	0.023 (0.014–0.04)	0.11^b^ (0.08–0.63)	2	0.08^b^ (0.1–0.16)	0	0.084^c^ (0.048–0.095)	0	0.1^b^ (0.08–0.16)	2	0.65^a^ (0.35–3.1)	5
	GGT:Crea (< 5 IU/mmol)	1.07 (0.74–1.52)	1.9^b^ (1.5–2.2)	0	1.35 (1.25–1.56)	0	1.31 (1.12–1.54)	0	1.44 (1.22–1.97)	0	4.55^ac^ (1.62–7.19)	5
FENa (< 1%)		0.08 (0.06–0.12)	0.14 (0.13–0.15)	0	0.1^c^ (0.06–0.16)	1	0.12^c^ (0.05–0.23)	0	0.13 (0.07–0.17)	1	1.95^ac^ (0.44–5.8)	6

Q1, quartile 1; Me, median; Q3, quartile 3; S, serum; U, urine; Crea, creatinine; SG, specific gravity; UPCR, urine protein creatinine ratio; GGT, Gamma-glutamyltransferase; FENa, Fractional sodium excretion. n’, number of horses with value outside the laboratory reference value (parentheses in the first column) [4]. ^a^significant differences between non-AKI and AKI group; ^b^significant differences between non-AKI and high-risk group; ^c^significant differences between AKI-risk and AKI group

Table 2 shows SDMA levels determined in non-AKI, high-risk and AKI groups of animals. There were no significant differences in median SDMA concentrations between the non-AKI and high-risk groups. SDMA levels, however, were significantly higher in horses with AKI than in the two other groups.

**Table 2:** Serum symmetric dimethylarginine (SDMA) measured in healthy horses (non-AKI group), horses at risk of acute kidney injury (high-risk group; divided into horses with colic and horses treated with NSAIDs and gentamicin), and horses with clinically manifest acute kidney injury (AKI group)

Parameter	Non-AKI group (n = 30)			High-risk group (n = 30)												AKI group (n = 11)		
				Colic (n = 10)			NSAIDs (n = 10)			Gentamicin (n = 10)			All					
	Min–max	Me (Q1–Q3)	n’	Min–max	Me (Q1–Q3)	n’	Min–max	Me (Q1–Q3)	n’	Min–max	Me (Q1–Q3)	n’	Min–max	Me (Q1–Q3)	n’	Min–max	Me (Q1–Q3)	n’
SDMA (µg/dL)	10–18	12 (11–14)	2	6–18	12.5 (10.25–14.5)	2	9–14	11 (9.25–11.75)	0	12–15	13 (12–13.75)	0	6–18	12 (11–13)	2	19–62	20 (20–37)	11

Q1, quartile 1; Me, median; Q3, quartile 3. n’, number of horses with values above the upper normal range (> 14 µg/dL) [26]

The correlation coefficients obtained in the analysis of SDMA as well as selected serum and urine parameters in the non-AKI and AKI horses are shown in Table 3. There was no effect of age or sex on serum SDMA concentrations. There was a medium positive correlation between SDMA and the serum creatinine levels, a low positive correlation between SDMA and BUN levels and a low negative correlation between SDMA level and specific gravity determined in urinalysis.

**Table 3:** Spearman correlation coefficients for the associations between the SDMA level and selected parameters in all enrolled horses

	Body weight	Serum		Urine					FENa
		Urea	Creatinine	pH	SG	Protein	UPCR	GGT:Crea	
SDMA	NS	r_s_ = 0.33 P = 0.005	r_s_ = 0.48 P < 0.001	NS	r_s_ = −0.35 P < 0.005	r_s_ = 0.24 P < 0.05	NS	NS	NS

r_s_, Spearman correlation coefficient; SG, specific gravity; UPCR, urine protein to creatinine ratio; GGT:Crea–urine gamma-glutamyltransferase to creatinine ratio; FENa, Fractional sodium excretion; NS, non-significant

**Fig. 1:**
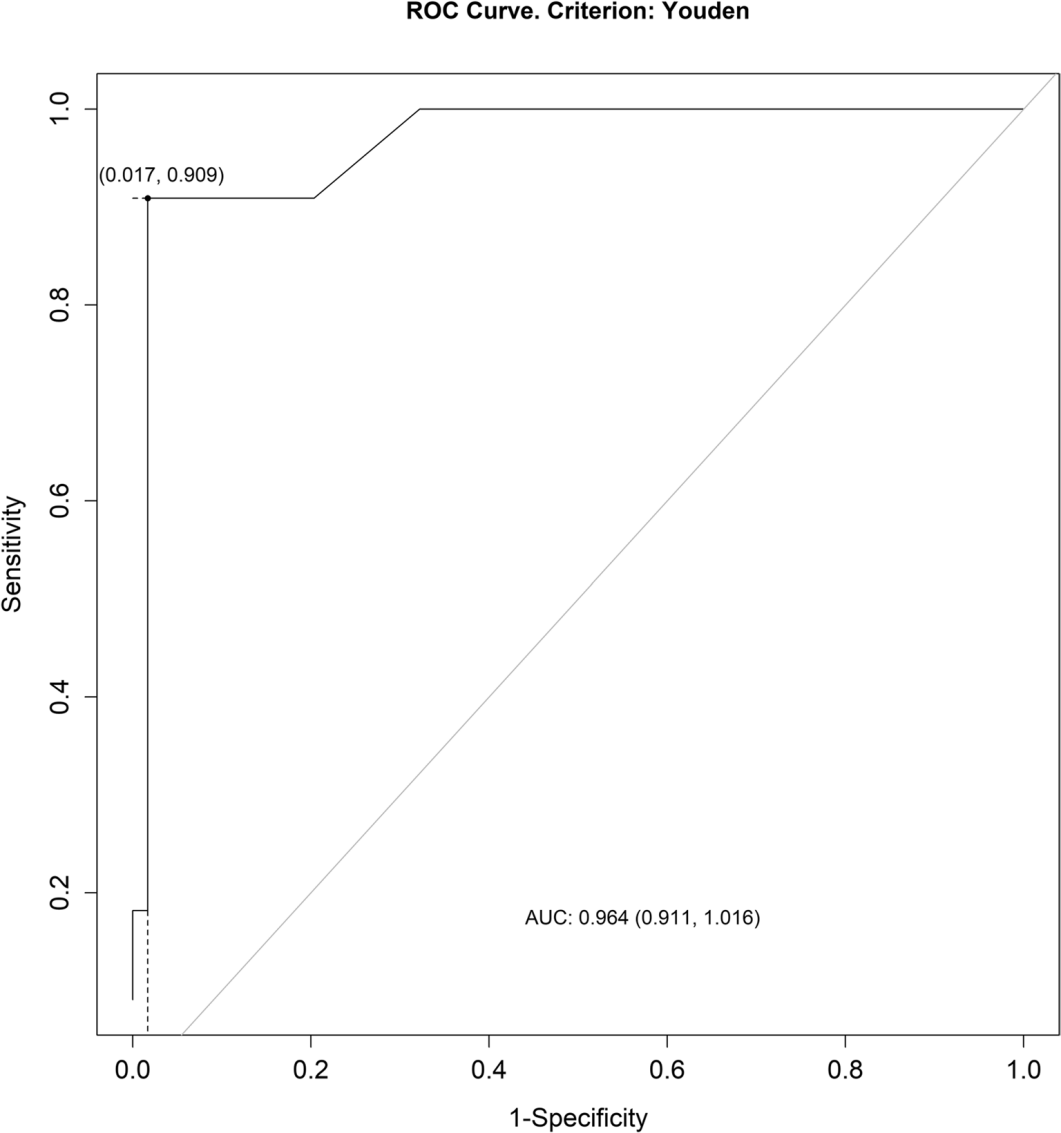
A ROC curve showing the sensitivity and specificity of SDMA in detecting acute kidney dysfunction in enrolled horses

Table 4 and Fig. 1 present the ROC curve plotted for SDMA assay performance in detecting acute kidney injury in enrolled horses.

**Table 4:** The ROC analysis of SDMA assay performance in detecting AKI in horses

Cut-off value	Sensitivity	Specificity	PPV	NPV	AUC
19.0	0.91	0.98	0.91	0.98	0.96

AUC, area under the ROC curve, PPV, positive predictive value, NPV, negative predictive value

The optimal cut-off value for SDMA was 19. The assay proved to be more effective detecting negative than positive results. The SDMA ability to differentiate between normal and abnormal results was good (0.96).

## Discussion

The current study assessed serum SDMA levels and their correlation with conventional biomarkers of kidney function in horses with AKI, those at risk of developing AKI, and healthy ones. To the best of authors’ knowledge, this is the first study to assess serum SDMA concentration at pre-clinical stages of AKI in horses with gastrointestinal disease and horses treated with potentially nephrotoxic drugs. Furthermore, only a few reports have been published on SDMA levels in horses, none of which report studies carried out in animals at risk of developing AKI [26–29].

SDMA, a biomarker enabling early detection of impaired kidney function, is routinely used in human and companion animal medicine. It is an endogenous methylated arginine released into the bloodstream during normal protein catabolism [22]. Physiologically, 90% of SDMA is excreted in an unchanged form with urine [19, 23, 32]. However, it is accumulated in the body in kidney failure [33].

The only report assessing SDMA concentrations in adult horses indicated that the normal ranges in healthy horses are similar to those seen in companion animals with an upper limit of 14 µg/dL [26]. In our study, though, two horses from the non-AKI group had an SDMA level above this threshold value. Subclinical AKI seems unlikely in these two horses, as other parameters remained unchanged and they did not develop kidney disease. This conclusion seems consistent with findings of Hall et al. [24], who identified two false-positive cases of SDMA elevation without an accompanying reduced GRF in cats. Nevertheless, a transient renal impairment cannot be reliably excluded due to the lack of sensitive and validated diagnostic tools for the detection of early AKI in equines.

The cut-off value of SMDA concentration of 19 µg/dL assumed in our study was higher than previously reported. As it was the case in the non-AKI group, all but two horses from the high-risk group in our study had SDMA concentrations within the normal range. Whereas serum creatinine levels in these two horses were within normal range, urinalysis revealed some abnormalities. The SDMA assay may have enabled us to detect early renal impairment, yet neither of these horses developed clinically manifest AKI. It appears that using SDMA assay as a stand-alone biomarker may be insufficient in clinical practice due to the risk of false-positive results and a higher SDMA cut-off value in horses.

All horses with AKI had elevated SDMA concentrations with the median SDMA level significantly higher compared to other groups. The SDMA levels in horses with AKI were similar to those obtained in humans and companion animals, where they were shown to be even four times higher than in healthy individuals [19, 21, 24, 25]. Horses with higher serum creatinine levels also had higher SDMA concentrations, which may indicate a positive relationship between the SDMA level and the severity of renal impairment reported in other species [34]. Considering the absence of the false-negative results in the AKI group and the ability of the SDMA assay to distinguish between horses with and without azotaemia, it may be a useful biomarker of clinical renal impairment. On the other hand, the utility of SDMA assay in subclinical cases is limited by the possible false-positive results whereby AKI is associated with structural damage to the kidney rather than its functional impairment.

Renal complications of gastrointestinal diseases and side-effects of potentially nephrotoxic drugs have been well documented in horses [8–14]. In our study, we observed abnormally elevated urinalysis parameters in the high-risk group, despite the lack of systemic involvement and serum creatinine and BUN elevation. Horses with colic and horses treated with NSAIDs had higher urinary protein and UPCR levels than the healthy animals enrolled in our study. Arosalo et al. [8] also found horses with colic to have elevated UPCR levels. Proteinuria, which accompanies glomerular and tubular injury, may indicate AKI. In humans, proteinuria indicates tubular necrosis and constitutes a stand-alone treatment indication [35]. In horses, though, transient proteinuria can be associated with inflammation or haemorrhage (e.g., post-exercise) [3, 4, 36]. Horses with colic enrolled in our study also had an elevated urinary GGT:Crea ratio, as reported by Arosalo et al. [8]. The increased activity of GGT, a constitutive enzyme of the proximal tubular brush-border, is reflected by elevated urinary GGT:Crea ratio, which supports the hypothesis of its association with tubular injury [4]. The elevation of several urinary biomarkers in horses with colic may indicate early-stage kidney injury and/or impairment. The reduced GFR in horses with gastrointestinal disease can be explained by the haemodynamic changes [37]. On the other hand, the urinary GGT:Crea ratio should be interpreted with caution, as its spikes are often transient and reversible in horses treated for various infections [3, 12, 38]. Some of those horses received treatment and underwent general anaesthesia prior to urine sampling, which could have affected the results.

The serum and urinary parameters remained within the normal range in the enrolled horses treated with gentamicin, which stands in contrast with frequent reports of gentamicin-induced AKI [11, 12, 38]. Our equine subjects received gentamicin at the recommended dose once daily for a short period of time, they were all free of any systemic disease, which limits possible side effects known to occur in juvenile animals with multi-organ disease [11, 39].

The current study had some limitations. First of them is sample heterogeneity, both in high-risk group and in the AKI group (multiple aetiologies). Animals in both groups had comorbidities, which may have influenced the study results. However, such situations are is not uncommon of in real-life clinical practice, where animals develop AKI due to different primary diseases [2]. Since AKI encompasses both structural damage to the kidney and its dysfunction, optimum assessment of AKI may ultimately require a panel of different biomarkers. However, further extensive research into the expression of selected biomarkers, in both healthy and diseased animals, is needed before they can be routinely used in equine medicine. The second limitation of the study was the lack of histologic evaluation of kidney biopsy specimens, GRF determination and its correlation with biomarker levels, which limited the objectivity of kidney function assessment. We used serum creatinine concentration as the current gold standard primarily due to the lack of available alternatives. Unfortunately, using serum creatinine level as a marker of kidney function cannot totally exclude early-stage AKI. Furthermore, the effect of non-renal factors on creatinine levels may further limit its use. Finally, the study involved the determination of SDMA levels and other conventional biomarkers of kidney function at a single time-point, limiting the possibility to track their temporal changes.

## Conclusion

The SDMA cut-off value obtained in healthy horses was higher than the one reported by others. The SDMA assay correctly identified all horses with clinically manifest acute kidney dysfunction. Due to a positive correlation between the SDMA level and azotaemia, SDMA was recognised as useful in more advanced cases of AKI to confirm kidney dysfunction. However, its utility may be limited in subclinical AKI. Abnormalities limited to a single urine parameter were seen in horses at risk of developing AKI alongside normal SDMA levels, suggesting higher efficacy of urinary markers in detecting early kidney dysfunction. More numerous and significant abnormal findings were seen in horses with colic, which may indicate that the impact of gastrointestinal conditions on kidney function is more significant than the one associated with routine use of potentially nephrotoxic drugs.

## Data Availability

All data generated or analysed during this study has been made available in this article.

## Abbreviations

AKI: Acute kidney injury
ALT: Alanine aminotransferase
AST: Aspartate aminotransferase
BUN: Blood urea nitrogen
CK: Creatine kinase
FENa: Fractional excretion of sodium
GFR: Glomerular filtration rate
GGT: Gamma-glutamyl transpeptidase
GGT:Crea: Urinary GGT to creatinine ratio
NPV: Negative predictive value
NSAIDs: Non-steroidal anti-inflammatory drugs
PPV: Positive predictive value
r_s_: Spearman correlation coefficient
SDMA: Symmetric dimethylarginine
SG: Specific gravity
SIRS: Systemic inflammatory response syndrome
TP: Total protein
UPCR: Urine protein to creatinine ratio

## References

[1] Levey AS, James MT (2017). Acute kidney injury. Ann Intern Med.

[2] Makris K, Spanou L (2016). Acute kidney injury: definition, pathophysiology and clinical phenotypes. Clin Biochem Rev.

[3] Geor RJ (2007). Acute renal failure in horses. Vet Clin North Am Equine Pract.

[4] Schott HC, Esser MM (2020). The sick adult horse: renal clinical pathologic testing and urinalysis. Vet Clin North Am Equine Pract.

[5] Mehta RL, Kellum JA, Shah SV, Molitoris BA, Ronco C, Warnock DG (2007). AKIN. Acute kidney injury network: report of an initiative to improve outcomes in acute kidney injury. Crit Care..

[6] Savage VL, Marr CM, Bailey M, Smith S (2019). Prevalence of acute kidney injury in a population of hospitalized horses. J Vet Intern Med.

[7] Groover ES, Woolums AW, Cole DJ, LeRoy BE (2006). Risk factors associated with renal insufficiency in horses with primary gastrointestinal disease: 26 cases (2000–2003). JAVMA.

[8] Arosalo BM, Raekallio M, Rajamäki M, Holopainen E, Kastevaara T, Salonen H (2007). Detecting early kidney damage in horses with colic by measuring matrix metalloproteinase -9 and -2, other enzymes, urinary glucose and total proteins. Acta Vet Scand.

[9] Divers TJ, Whitlock RH, Byars TD, Leitch M, Crowell WA (1987). Acute renal failure in six horses resulting from hemodynamic causes. Equine Vet J.

[10] Seanor JW, Byars TD, Boutcher JK (1984). Renal disease associated with colic in horses. Mod Vet Pract.

[11] Sweeney RW, Macdonald M, Hall J, Divers TJ, Sweeney CR (1988). Kinetics of gentamicin elimination in two horses with acute renal failure. Equine Vet J.

[12] van der Harst MR, Bull S, Laffont CM, Klein WR (2005). Gentamicin nephrotoxicity–a comparison of in vitro findings with in vivo experiments in equines. Vet Res Commun.

[13] Collins LG, Tyler DE (1984). Phenylbutazone toxicosis in the horse: a clinical study. J Am Vet Med Assoc.

[14] MacAllister CG, Morgan SJ, Borne AT, Pollet RA (1993). Comparison of adverse effects of phenylbutazone, flunixin meglumine, and ketoprofen in horses. J Am Vet Med Assoc.

[15] Bellomo R, Ronco C, Kellum JA, Mehta RL, Palevsky P (2004). Acute renal failure–definition, outcome measures, animal models, fluid therapy and information technology needs: the Second International Consensus Conference of the Acute Dialysis Quality Initiative (ADQI) Group. Crit Care.

[16] Grossman BS, Brobst DF, Kramer JW, Bayly WM, Reed SM (1982). Urinary indices for differentiation of prerenal azotemia and renal azotemia in horses. JAVMA.

[17] El-Ashker MR, Hussein HS, El-Sebaei MG (2012). Evaluation of urinary variables as diagnostic indicators of acute kidney injury in Egyptian draft horses treated with phenylbutazone therapy. J Eq Vet Sci.

[18] Jacobsen S, Berg LC, Tvermose E, Laurberg MB, van Galen G (2018). Validation of an ELISA for detection of neutrophil gelatinase-associated lipocalin (NGAL) in equine serum. Vet Clin Pathol.

[19] Schwedhelm E, Boger RH (2011). The role of asymmetric and symmetric dimethylarginines in renal disease. Nat Rev Nephrol.

[20] Zhang WR, Parikh CR (2019). Biomarkers of acute and chronic kidney disease. Annu Rev Physiol.

[21] Fleck C, Schweitzer F, Karge E, Busch M, Stein G (2003). Serum concentrations of asymmetric (ADMA) and symmetric (SDMA) dimethylarginine in patients with chronic kidney diseases. Clin Chim Acta.

[22] Paltrinieri S, Giraldi M, Prolo A, Scarpa P, Piseddu E, Beccati M (2018). Serum symmetric dimethylarginine and creatinine in Birman cats compared with cats of other breeds. J Feline Med Surg.

[23] Kielstein JT, Boger RH, Bode-Boger SM, Froelich JC, Haller H, Ritz E (2002). Marked increase of asymmetric dimethylarginine in patients with incipient primary chronic renal disease. J Am Soc Nephrol.

[24] Hall JA, Yerramilli M, Obare E, Yerramilli M, Jewell DE (2014). Comparison of serum concentrations of symmetric dimethylarginine and creatinine as kidney function biomarkers in cats with chronic kidney disease. J Vet Intern Med.

[25] Dahlem DP, Neiger R, Schweighauser A, Francey T, Yerramilli M, Obare E (2017). Plasma symmetric dimethylarginine concentration in dogs with acute kidney injury and chronic kidney disease. J Vet Intern Med.

[26] Schott HC, Gallant L, Coyne M (2018). Symmetric dimethylarginine and creatinine concentrations in draft horse breeds. J Vet Intern Med.

[27] Bozorgmanesh R, Coyne M, Murphy R, Halliwell E, Coyne M, Murphey R, Hegarty E, Slovis N (2019). Equine neonatal symmetric dimethylarginine (SDMA): results of two pilot studies. J Vet Intern Med.

[28] Bozorgmanesh R, Magdesian G, Offer K, Hegarty E, Slovis N (2019). Equine neonatal symmetric dimethylarginine in sick neonates with hypercreatininemia. J Vet Emerg Crit Care.

[29] Siwinska N, Zak A, Slowikowska M, Niedzwiedz A, Paslawska U (2020). Serum symmetric dimethylarginine concentration in healthy horses and horses with acute kidney injury. BMC Vet Res.

[30] Siwińska N, Pasławska U, Bąchor R, Szczepankiewicz B, Żak A, Grochowska P (2020). Evaluation of podocin in urine in horses using qualitative and quantitative methods. PLoS ONE.

[31] Thoen ME, Kerl ME (2011). Characterization of acute kidney injury in hospitalized dogs and evaluation of a veterinary acute kidney injury staging system. J Vet Emerg Crit Care.

[32] Nijveldt RJ, Teerlink T, Van Guldener D, Prins HA, van Lambalgen AA, Stehouwer CD (2003). Handling of asymmetrical dimethylarginine and symmetrical dimethylarginine by the rat kidney under basal conditions and during endotoxemia. Nephrol Dial Transplant.

[33] Vallance P, Leone A, Calver A, Collier J, Moncada S (1992). Accumulation of an endogenous inhibitor of nitric oxide synthesis in chronic renal failure. Lancet.

[34] Pelander L, Häggström J, Larsson A, Syme H, Elliott J, Heiene R (2019). Comparison of the diagnostic value of symmetric dimethylarginine, cystatin C, and creatinine for detection of decreased glomerular filtration rate in dogs. J Vet Intern Med.

[35] Herget-Rosenthal S, Poppen D, Hüsing J, Marggraf G, Pietruck F, Jakob HG (2004). Prognostic value of tubular proteinuria and enzymuria in nonoliguric acute tubular necrosis. Clin Chem.

[36] Schott HC, Hodgson DR, Bayly WM (1995). Haematuria, pigmenturia and proteinuria in exercising horses. Equine Vet J.

[37] Wig DA, Yamada T, Havley A, Norlen BJ, Nygien K, Ojteg G (1978). Model for disseminated intravascular coagulopathy: bacterial sepsis in rhesus monkeys. J Lab Din Med.

[38] Rossier Y, Divers TJ, Sweeney RW (1995). Variations in urinary gamma glutamyl transferase/urinary creatinine ratio in horses with or without pleuropneumonia treated with gentamicin. Equine Vet J..

[39] Gronwall R, Brown MP, Hobbs S (1988). Serum gentamicin concentrations and pharmacokinetics in 2-week-old pony foals after intramuscular administration. J Equine Vet Sci.

